# OpenUp! Creating a cross-domain pipeline for natural history data

**DOI:** 10.3897/zookeys.209.3179

**Published:** 2012-07-20

**Authors:** Walter G. Berendsohn, Anton Güntsch

**Affiliations:** 1 Department of Biodiversity Informatics and Laboratories, Botanic Garden and Botanical Museum Berlin-Dahlem, Freie Universität Berlin, Königin-Luise-Straße 6-8, D-14195 Berlin, Germany

**Keywords:** OpenUp!, BioCASe, EUROPEANA, GBIF, Multimedia, ABCD, ESE, EDM, Biodiversity Informatics, Collections, Natural History

## Abstract

Multimedia data held by Natural History Museums and Universities are presently not readily accessible, even within the natural history community itself. The EU project OpenUp! is an effort to mobilise scientific biological multimedia resources and open them to a wider audience using the EUROPEANA data standards and portal. The connection between natural history and EUROPEANA is accomplished using well established BioCASe and GBIF technologies. This is complemented with a system for data quality control, data transformation and semantic enrichment. With this approach, OpenUp! will provide at least 1,1 Million multimedia objects to EUROPEANA by 2014. Its lean infrastructure is sustainable within the natural history community and will remain functional and effective in the post-project phase.

## Introduction

**Figure 1.**
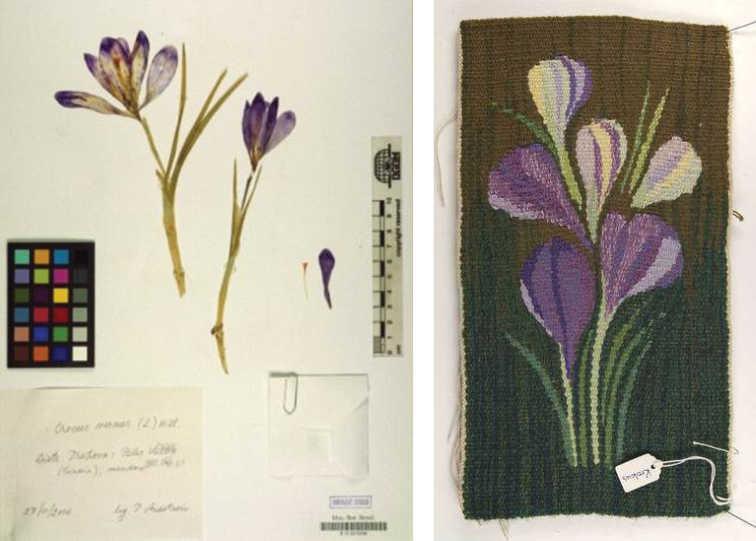
Herbarium specimen Crocus vernus L. (© Botanic Garden and Botanical Museum Berlin-Dahlem, Germany) and Tapestry called Krokus by Britta Rendahl (1976) (© Upplandsmuseet, Uppsala, Sweden).

The vast majority of global collections of biological organisms and images of organisms are held by institutions such as natural history museums and universities, in the realm of natural sciences. Nevertheless, nature is of course a major subject in the context of cultural history and humanities, and numerous cultural objects represent organisms (Fig. 1). Both communities have started to digitise their objects and to publish the resulting multimedia data to make them accessible to a wider audience. The prevalent disjunction between them, however, has led to procedures, technologies, and data standards being optimized for the respective community’s needs. The resulting incompatibilities prevent semantic linking and joined access.

In fact, there is a significant need for convenient joint access to the collection and multimedia holdings of different scientific communities. In the context of art history, for example, access to plant identifications provided by herbaria can be an important tool for the analysis of, e.g., ornaments in works of art. In turn, linking artwork with natural history specimens raises the general awareness of this important research tool and thus serves the museum community. And cultural background may be documented with natural history specimens; e.g. the collections during famous expeditions like those of Humboldt and Bonpland, and data on local uses recorded with the description of the collected organism.

EUROPEANA is the European portal to museums, libraries, archives, and audio-visual collections (Purday 2009). EUROPEANA has the potential to bridge the gulf between multimedia collections held by different communities by providing a common cross-domain user portal and web services based on unified metadata standards. During its first years of construction, EUROPEANA was clearly focused on cultural content, largely neglecting natural science objects. A series of biodiversity-related EU-projects such as STERNA (Sterna 2008), BHL-Europe (BHL-Europe 2009), Natural Europe (Natural Europe 2012), and OpenUp! (Berendsohn et al. 2011) widened EUROPEANA’s scope to include natural history content. OpenUP! is the instrument for mobilizing and providing high volumes of biological multimedia collection objects for EUROPEANA. By end of the project (March 2014), OpenUp! will have delivered access to at least 1,1 Million objects and their corresponding data and metadata. More importantly, OpenUp! implements a sustainable pipeline from natural history collections to EUROPEANA (and potentially to other portals using the EUROPEANA standards). Recent initiatives to further digitisation of specimens (e.g. in the context of the industrial-scale e-RECOLNAT project in France, digitising all French herbarium specimens; or the NSF-funded iDigBio initiative in the US) will bring massive amounts of such objects on line. Using the OpenUp! approach, collection holders can publish their metadata and image locations, making them available to a wide audience beyond the natural history community. This pipeline scales up and will continue to function and provide access to the rapidly growing stock of multimedia content held by natural history institutions.

Of course we are fully aware of the problems of semantic mapping of metadata, especially with the taxonomic concepts represented by the name (e.g. Geoffroy and Berendsohn 2003). However, though this (as most of the retrievable information on the Internet) is not satisfying from a scientific view, we still posit that exposing natural history object information to a hugely enlarged audience (as offered by EUROPEANA) will help both the data providers as well as the users. The former will gain by the raised awareness of their holdings and by drawing attention to their cultural context, the latter will (in many cases for the first time in their life) become aware that such collections exist. And as a major side effect of mobilising the information for various networks simultaneously, researchers can choose to access the information through other interfaces that are less fuzzy in that respect (e.g. Güntsch et al. 2009).

## The OpenUp! approach

**Figure 2.**
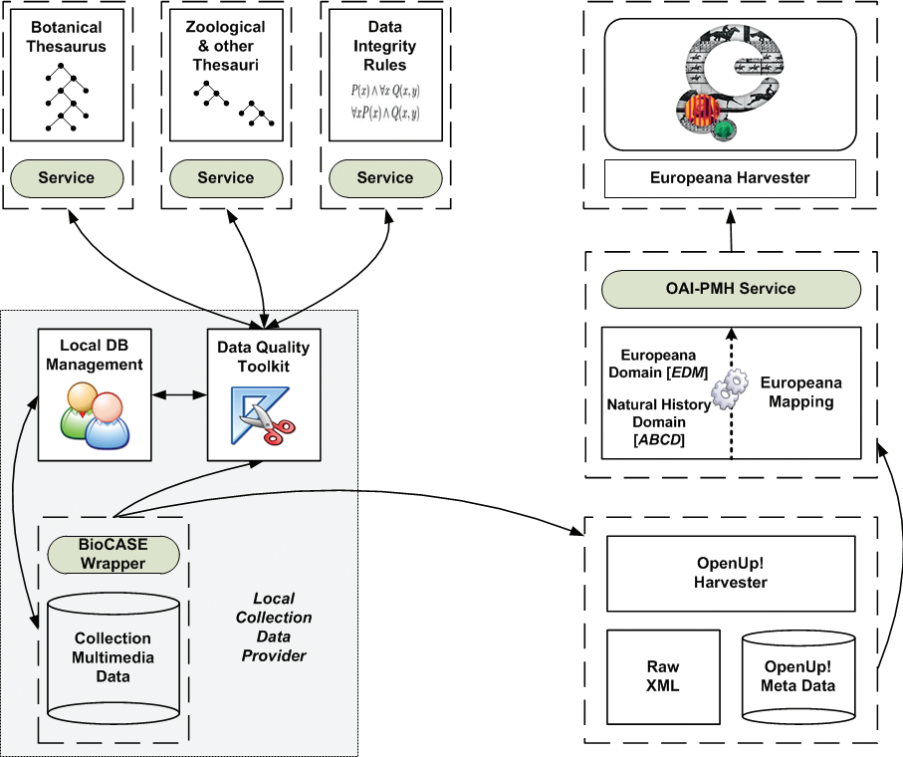
Information flow from a collection data provider via the central OpenUp! aggregator to the EUROPEANA harvester and portal. The collection database uses standard BioCASe/ABCD technology for connecting up to the network.

OpenUp! creates an information flow from holders of collection multimedia data to the EUROPEANA data portal and services, but it avoids as much as possible the development and deployment of project-specific software modules. Rather, existing and well established protocols, standards, and software tools are used, resulting in an infrastructure that can be maintained with low maintenance costs beyond the funded project phase (Fig. 2).

OpenUp! data providers are usually connecting their existing collection management databases to the network. These databases are part of their institutional work flow so that maintenance and updating is part of the institutional setup. Connection is accomplished by equipping the local database with an installation of the BioCASe provider software package (Holetschek et al. 2009), and by mapping the local data definitions to the TDWG Biodiversity Information Standard “Access to Biological Collection Information” (ABCD, Berendsohn 2005). The software translates the local data to ABCD and allows querying the database over the Internet. The same installation is also used to provide data to the GBIF network. The only difference is that the configuration of the provider software for OpenUp! has to ensure that a minimal set of data elements required by the EUROPEANA portal are made available. The central OpenUp! aggregator notifies providers if this condition has not been met.

Harvesting of ABCD data and storage on the central aggregation server is performed using the GBIF Harvesting and Indexing Toolkit (HIT, GBIF 2011). The aggregator database stores only the textual data, including the URIs of the multimedia data. It is implemented using the same system that is used by the BHL Europe project. From there, the data from the ABCD standard used by the natural history domain are transformed into ESE (ESE 2011), which is used as a cross-domain metadata standard in EUROPEANA. The transformation is carried out using Pentaho Data Integration (aka Kettle, Pentaho 2011). The mapping between ABCD and ESE concepts is based on a thorough analysis of both standards, considering the semantics of natural history data elements used in a cross-domain context (Theeten et al. 2012).

OpenUp! metadata are periodically harvested by EUROPEANA via a single OAI-PMH access point at the aggregator database. Previews of multimedia objects for presentation and queries in the EUROPEANA portal are generated by EUROPEANA from full object URLs given in the metadata. The object itself and its presentation (e.g. using an image server or streaming software for audio files) stay with the provider, who also retains full rights of the multimedia file. The existence of the file is checked during the ABCD/ESE conversion process. Additionally, the central OpenUp! server will cyclically check the links to multimedia files and warn data providers if files become unavailable. In case of enduring problems, the links metadata will be excluded from the process.

### Data Quality Control

Organising the basic information flow and data transformation process from biological multimedia collections to the EUROPEANA portal took considerable project resources. However, improving the content with regard to data quality and usability is the main item in the OpenUp! budget (which is co-funded by the European Union and the participants in the project). To support this process, some tools were implemented to support providers in the detection of data quality problems in their databases. Again, this “Data Quality Toolkit” mostly relies on existing systems and only a relatively lightweight interface layer is specific to OpenUp!

**Figure 3.**
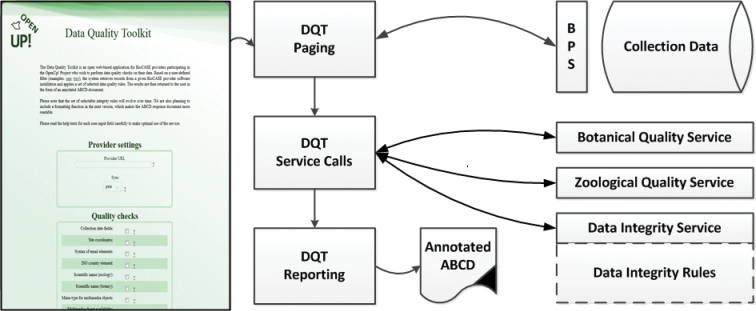
The OpenUp! Data Quality Toolkit

**Figure 4.**
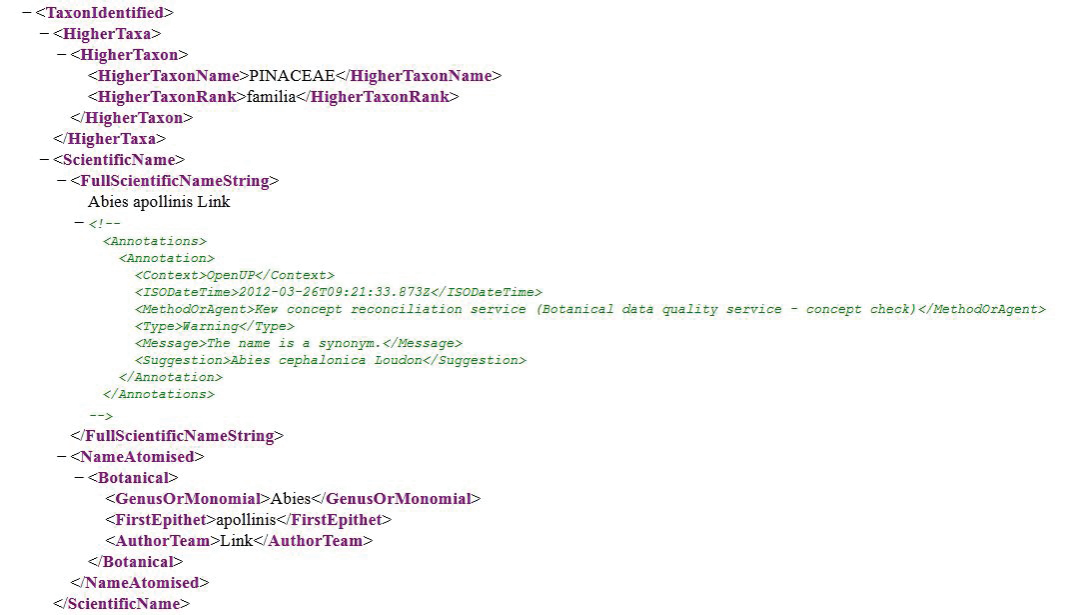
OpenUp! Data Quality Toolkit annotation indicating that an identification is using a name which is a synonym (according to a concept reconciliation service provided by Kew Gardens).

The OpenUp! Data Quality Toolkit (Fig. 3) operates directly on a given individual installation of the BioCASE provider software. It pages through a subset of ABCD records defined in its web-based user interface (OpenUp! 2012). Based on the user’s choice of data quality rules to be applied, ABCD elements are then sent to an evolving set of data quality services analysing particular aspects of the data. This includes botanical and zoological name and concept checks for identifications, checks of compliance of ABCD elements to controlled vocabularies (e.g. country codes, mime types for multimedia objects), and syntax of email-elements, dates and URLs. The toolkit then writes potential data problems as XML-encoded annotations directly into the ABCD-records they refer to and sends the compilation of all problem-records back to the user (Fig. 4). Users may also choose asynchronous access to avoid waiting periods. The tool provides suggestions to providers, which they may (or may not) take up in their OpenUp! quality enhancement task.

By decoupling the Data Quality Toolkit user interface layer from the underlying data quality services, the services themselves can be used in other contexts, and in turn, OpenUp! can integrate data quality services provided by other projects or initiatives. Collaborations have already started with the EU project BioVeL (Biodiversity Virtual e-Laboratory, BioVeL 2012) and the reBiND project funded by the German research foundation (Güntsch and Berendsohn in press).

### Semantic Enrichment

The impact of the presentation of natural history specimens in a cross-domain context like EUROPEANA will partly depend on the possibilities for semantic linking with other content. Semantic linking is made possible by the metadata provided, so it can be enhanced by enriching the domain vocabularies used by the providers in the metadata. For example, in natural history databases typically the Latin scientific name is entirely sufficient (and indeed the most precise way) to denote the identification of the specimen. In contrast, content from the cultural domain will usually refer to an organism by means of a common name. Users from that domain would not find the corresponding natural history object with their searches. Enhancing the natural history metadata by adding common names will close that gap.

In OpenUp! the botanical and zoological name services will be used to add synonym lists to the Latin names provided by the collection holders. A forthcoming OpenUp! service will be used for adding multilingual common names to the scientific names. In addition, external services will be used for adding further geographic information to the place names contained in the specimen data.

### Outlook

During the first project year, OpenUp! has mobilised more than 220,000 natural history multimedia objects and made them available through EUROPEANA and GBIF, and the numbers are rapidly growing. Specimens displayed in the EUROPEANA portal demonstrate the feasibility of the principle data flows in OpenUp!. However, they also brought to light the weakness of the portal or in fact of the underlying ESE standard. Multimedia objects representing collection objects often have a strong relation to each other (e.g. several images from one specimen), which the portal does not adequately represent in its present stage. With the transition to the new metadata standard EDM (Europeana Data Model, Doerr et al. 2010) planned for 2012, nested object structures will be implemented. The millions of objects expected from the Natural History world will provide an ideal test bed for both metadata for linked objects and portal user interfaces and services providing searchable access to complex structured data.
